# Suitable Stereoscopic Configuration of Electrolyte Additive Enabling Highly Reversible and High—Rate Zn Anodes

**DOI:** 10.3390/molecules29143416

**Published:** 2024-07-21

**Authors:** Binrui Xu, Yong Liu, Bo Zhao, Haoming Li, Min Liu, Huanxiao Mai, Quanan Li

**Affiliations:** 1 School of Information Engineering, Henan University of Science and Technology, Luoyang 471023, China; 9906065@haust.edu.cn (B.X.); liuimin1086@163.com (M.L.); mhxgby@163.com (H.M.); 2 School of Materials Science and Engineering, Provincial and Ministerial Coconstruction of Collaborative Innovation Center for Non—Ferrous Metal New Materials and Advanced Processing Technology, Henan University of Science and Technology, Luoyang 471023, China; bozhao123@haust.edu.cn (B.Z.); lihaoming546123@gmail.com (H.L.); 3 Key Laboratory of Cluster Science of Ministry of Education, Beijing Key Laboratory of Photoelectronic/Electrophotonic Conversion Materials, School of Chemistry and Chemical Engineering, Beijing Institute of Technology, Beijing 100081, China; 4 Longmen Laboratory, Luoyang 471000, China

**Keywords:** Zn anode, electrolyte additive, stereoscopic configuration, dendrite growth, side reactions

## Abstract

Electrolyte additive engineering is a crucial method for enhancing the performance of aqueous zinc—ion batteries (AZIBs). Recently, most research predominantly focuses on the role of functional groups in regulating electrolytes, often overlooking the impact of molecule stereoscopic configuration. Herein, two isomeric sugar alcohols, mannitol and sorbitol, are employed as electrolyte additives to investigate the impact of the stereoscopic configuration of additives on the ZnSO_4_ electrolyte. Experimental analysis and theoretical calculations reveal that the primary factor for improving Zn anode performance is the regulation of the solvation sheath by these additives. Among the isomers, mannitol exhibits stronger binding energies with Zn^2+^ ions and water molecules due to its more suitable stereoscopic configuration. These enhanced bindings allow mannitol to coordinate with Zn^2+^, contributing to solvation structure formation and reducing the active H_2_O molecules in the bulk electrolyte, resulting in suppressed parasitic reactions and inhibited dendritic growth. As a result, the zinc electrodes in mannitol—modified electrolyte exhibit excellent cycling stability of 1600 h at 1 mA cm^−2^ and 900 h at 10 mA cm^−2^, respectively. Hence, this study provides novel insights into the importance of suitable stereoscopic molecule configurations in the design of electrolyte additives for highly reversible and high—rate Zn anodes.

## 1. Introduction

In contrast to traditional lithium—ion batteries utilizing organic electrolytes, rechargeable aqueous zinc—ion batteries (AZIBs) are being increasingly recognized as a promising replacement for energy storage devices by virtue of their cost effectiveness, reliable security, eco—friendliness, and potentially high power density of zinc metal [1,2,3,4,5,6,7,8]. Nevertheless, the practical application of AZIBs faces significant challenges due to the poor reversibility of the zinc anode during the charge/discharge process in the aqueous electrolyte [9,10,11,12]. The coordinated water within the solvation shell of the Zn^2+^ ion and the active H_2_O in the aqueous electrolyte trigger severe parasitic reactions on the zinc metal surface, including corrosion and generation of H_2_ gas, thereby producing the by—products and exacerbating the inhomogeneous deposition of Zn^2+^ [13,14,15,16]. As such, this inevitably leads to a decline in the plating/stripping Coulombic efficiency (CE) and eventually to short circuits, cell bulging, and explosion [17]. 

Various approaches have been employed to address the mentioned shortcomings of AZIBs, including the manufacture of alloy anode, fabrication of coating layer, design of three—dimensional host, development of innovative electrolyte, and electrolyte modification [18,19,20,21,22,23,24,25,26]. In particular, modifying the electrolyte stands out as one of the most expedient solutions to advance the commercial viability of AZIBs, owing to its excellent simplicity, reproducibility, and versatility. Current research is primarily focused on the development of electrolyte additives that are based on the regulation of Zn^2+^—solvation structure and the modification of the interface between the aqueous electrolyte and Zn anode, such as salts, polymers, nanoparticles, and organic molecules [27,28,29,30,31,32]. For instance, Feng et al. demonstrated that the introduction of DMSO molecule into ZnSO_4_ electrolyte reduces side reactions in the Zn^2+^—solvation structure caused by H_2_O molecules, optimizes Zn^2+^ nucleation by texturing the (002) plane, and facilitates fine—grained deposition to enhance resistance to side reactions and dendrite formation [33]. Additionally, Guo et al. introduced dopamine as an electrolyte additive, which can adhere to the Zn anode surface to form a protective layer, thereby enhancing the cycling stability of Zn anode to 1000 h under 1 mA cm^−2^ and 1 mAh cm^−2^ [34]. Moreover, Hu et al. proposed an innovative xylitol additive that inhibits the hydrogen evolution reaction (HER), expels active H_2_O molecules, accelerates cations migration, and weakens electrostatic interaction through oriented restructuring of hydrogen bonds. The Zn/Zn symmetrical cell utilizing the xylitol—modified electrolyte retained reversible plating/stripping for over 1100 h at 1 mA cm^−2^ and 1 mAh cm^−2^ [35]. Furthermore, Wang et al. utilized ethylene glycol to tune Zn^2+^ coordination environment, thus suppressing detrimental dendrite growth on Zn anode and extending the cycling lifespan to 2668 h at 0.5 mA cm^−2^ and 0.5 mAh cm^−2^ [36]. 

Although the above—mentioned additives significantly extend the electrochemical performance of zinc electrodes by the regulation of the coordination environment of Zn^2+^ or the modification of the electrolyte—electrode interface, the cycling stability of AZIBs deteriorates sharply under severe test conditions (>2 mA cm^−2^, >2 mAh cm^−2^), making them impractical for real—world applications [37,38]. Hence, it is crucial to design efficient additives that address the existing obstacles of AZIBs and satisfy practical needs, especially under harsh test conditions. Furthermore, while the existing research predominantly focuses on the impact of the polar functional groups in these additives, the impact of the molecule stereoscopic configuration of additives is often overlooked. Isomers, compounds with identical molecule formulas and functional groups but different stereoscopic configurations, possess distinct chemical and physical properties, likely influencing their effectiveness as electrolyte additives. Despite this, investigations into the role of stereoscopic configurations on additive performance remain limited, highlighting a significant obstacle in designing efficient electrolyte additives for AZIBs.

Herein, a pair of isomers, mannitol and sorbitol, were incorporated as additives into the aqueous ZnSO_4_ electrolyte to investigate the impact of the stereoscopic configuration of additive molecules on the performance of AZIBs. Mannitol and sorbitol, despite sharing a similar structure as sugar alcohols with six hydroxyl groups, possess distinct spatial configurations due to the chirality of a carbon atom within the molecules. Experimental analyses and theoretical computations revealed that sugar alcohol molecules displace the coordinated H_2_O molecules within the Zn^2+^—solvation structure, thus restraining the parasitic reactions caused by H_2_O on the Zn electrode surface. In addition, the strong binding energy between H_2_O and sugar alcohol leads to a reduction in free water surrounding the solvation shell, thereby effectively mitigating spontaneous parasitic reactions and suppressing dendritic growth. The research findings also indicate that the stereoscopic configuration of mannitol is more suitable for the ZnSO_4_ electrolyte, resulting in Zn/Zn cells exhibiting stable cycling performance for more than 1600 h at 1 mAh cm^−2^ (1 mAh cm^−2^) and over 900 h even under the harsh condition of 10 mA cm^−2^ (10 mAh cm^−2^). Therefore, this study suggests a new direction in the design of electrolyte additives, highlighting the critical role of the stereoscopic configuration of additive molecules in regulating the solvation structure of electrolytes.

## 2. Results and Discussions

### 2.1. Electrolytes’ Characterization

Mannitol and sorbitol, which are isomers of each other, possess six hydroxyl groups that have a strong affinity with charged metal ions, and the stereoscopic configurations of these molecules are shown in Figure S1 [35]. To determine the optimal concentration of sugar alcohol as an electrolyte additive, the cyclic performances of symmetrical Zn/Zn cells with different concentrations of mannitol were measured, as illustrated in Figure S2. Among the tested concentrations, the Zn/Zn cell using a 2 M ZnSO_4_ electrolyte with 20 mM mannitol exhibited the longest cycling lifespan. Therefore, in order to examine the influence of the stereoscopic configuration of sugar alcohols on the ZnSO_4_ electrolyte, 20 mM mannitol and sorbitol were individually dissolved in 2 M ZnSO_4_ aqueous electrolyte (Figure S3) and denoted as ZnSO_4_@M and ZnSO_4_@S. 

**Figure 1:**
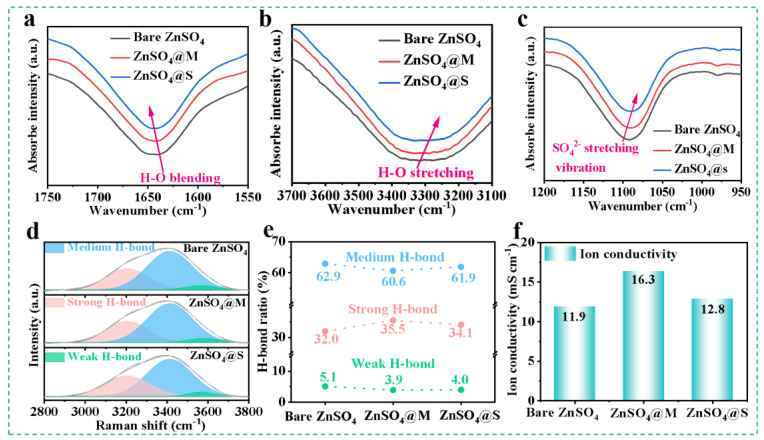
FT—IR spectra of the (**a**) H—O blending vibration, (**b**) H—O stretching vibration, and (**c**) SO_4_^2−^ stretching vibration of ZnSO_4_ electrolytes with/without additive. (**d**) Raman spectra (H—O stretching) of the different electrolytes. (**e**) The percentages of strong, medium, and weak H—bonds in the different electrolytes. (**f**) Ionic conductivities of the different electrolytes.

Fourier transform infrared spectrometer (FT—IR)was conducted to practically investigate the regulation of the solvation structure of Zn^2+^ by sugar alcohol additives [39]. Figure 1a,b show that the H—O bending vibration shifts towards higher wavenumbers and the H—O stretching vibration shifts towards lower wavenumbers in the ZnSO_4_@M and ZnSO_4_@S electrolytes. This indicated that the addition of sugar alcohols led to the displacement of H_2_O molecules from the Zn^2+^—solvation sheath into the bulk solution phase, augmenting the number of hydrogen bonds [36]. This displacement mechanism suggests that sugar alcohols can coordinate with Zn^2+^ and modify the coordination environment in the ZnSO_4_ electrolyte [40]. Additionally, the stretching vibration of SO_4_^2−^ in the electrolyte shifted upon the introduction of sugar alcohols, indicating a loosened constraint around SO_4_^2−^ and highlighting the reconstruction of the Zn^2+^—solvation structure due to the additives [41].

The electrolyte modification is further determined by the Raman spectroscopy analysis [42]. The Raman spectra of ZnSO_4_@M and ZnSO_4_@S electrolytes (Figure S4) show a new peak of C=C vibration (1600–1700 cm^−1^), which is attributed to the incorporation of sugar alcohols. In addition, the broad peak of H—O stretching vibration (2800–3800 cm^−1^) was segmented into three different peaks (Figure 1d), each of which corresponds to strong, medium, and weak H—bonds, respectively [43]. Findings from the deconvoluted peak region in Figure 1e indicate that the addition of sugar alcohols, particularly mannitol, led to a decrease in the proportions of medium and weak hydrogen bonds at higher frequency and an increase in the percentage of strong H—bonds at lower frequency. Specifically, mannitol and sorbitol additives were found to strengthen hydrogen bonding interactions, leading to enhanced water cluster stability and reduced active H_2_O content in the ZnSO_4_ electrolyte [44,45]. Furthermore, the slight differences in the changes to hydrogen bonds induced by the two additives may be attributed to variations in their stereoscopic configurations. Moreover, the enhanced ionic conductivity observed in both ZnSO_4_@M and ZnSO_4_@S electrolytes (Figure 1f) implied that the modified coordination environment in the presence of additives contributed to a reduction in water activity [31]. Furthermore, Figure S5 illustrates that the cyclic voltammogram (CV) curves of Zn/Zn cell with ZnSO_4_@M electrolyte in multiple cycles aligned well, indicating the excellent electrochemical stability of mannitol additive [46].

### 2.2. Theoretical Calculation

**Figure 2:**
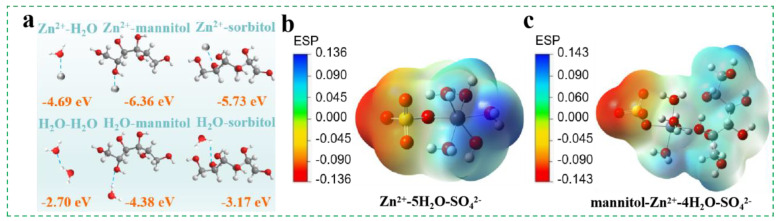
(**a**) The binding energy comparison of various pairs. ESP mapping of the (**b**) Zn^2+^–5H_2_O–SO_4_^2−^ and (**c**) mannitol—Zn^2+^—4H_2_O—SO_4_^2−^ solvation structures.

To further verify the effect of stereoscopic configuration on the coordination ability of the additive molecules, the binding energies of the Zn^2+^—H_2_O pair (−4.6 eV), Zn^2+^—mannitol pair (−6.36 eV), and Zn^2+^—sorbitol pair (−5.73 eV) were calculated using density functional theory (DFT), as illustrated in Figure 2a. The stronger interaction observed between the cationic Zn^2+^ and hydroxyl group from the sugar alcohol molecule suggests that the sugar alcohol preferentially participates in the formation of the Zn^2+^—solvation structure, which is also supported by the above FT—IR spectra [33]. In addition, Figure 2a also presents the binding energies of the mannitol—H_2_O pair, sorbitol—H_2_O pair, and H_2_O—H_2_O pair, where the mannitol—H_2_O and sorbitol—H_2_O pairs exhibit higher binding energies, particularly the mannitol—H_2_O pair. These DFT results indicate a tendency for the reconstruction of the water hydrogen bond network in the ZnSO_4_ electrolyte, wherein more free H_2_O molecules bind with sugar alcohol molecules, as supported by the consistent Raman spectra. Therefore, it can be speculated that the addition of sugar alcohol additive regulates the solvation structure of Zn^2+^ and restructures the H—bond composition in the electrolyte. Furthermore, the greater binding affinity of mannitol towards Zn ions and water molecules, compared to sorbitol, suggests that its steric configuration is more effective in influencing the solvation structure of the ZnSO_4_ electrolyte. 

Notably, Figure 2b,c exhibits that the electrostatic potential of the coordination complex increases when a water molecule is replaced by a sugar alcohol molecule, which results in a more uneven distribution. This uneven distribution helps alleviate electrostatic potential in the vicinity of Zn^2+^ and promotes the rapid transport of Zn^2+^. Specifically, sites with higher positive electrostatic potential are more appealing to negative sites, thereby favoring the directional reconstruction of H—bonds associated with the mannitol molecule [35]. Additionally, the theoretical geometric parameters of solvation structures are provided in Table S1 and Figure S6 to further elucidate the impact of mannitol additive on the solvation structure of Zn^2+^. Results show that the addition of mannitol causes a decrease in the average bond length of Zn^2+^—H_2_O and Zn^2+^—SO_4_^2−^ bond length, indicating that another part of the structure, apart from the mannitol molecule, becomes more compact, possibly hindering water molecules from participating in the solvation structure. This change in bond lengths also suggested that the symmetry of the entire structure weakens upon the addition of mannitol, making it more stable in an aqueous solution [47,48].

### 2.3. Cycling Performance

**Figure 3:**
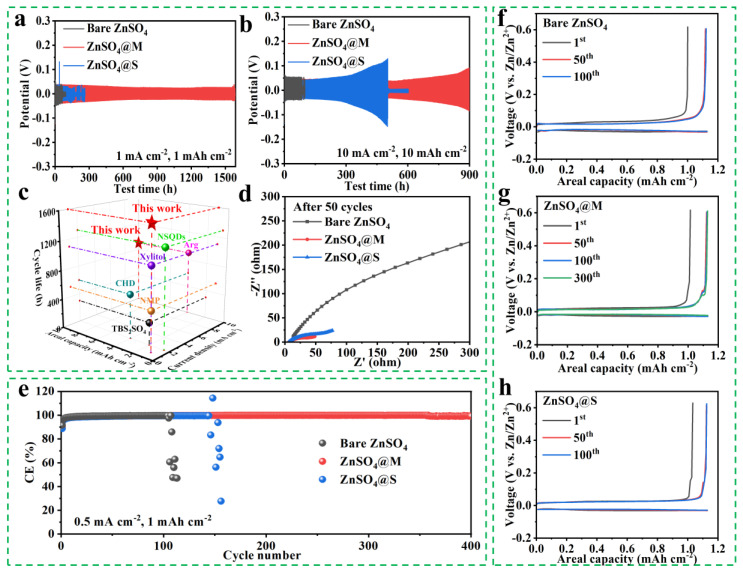
Cyclic performance of Zn/Zn cells with different electrolytes under (**a**) 1 mA cm^−2^ with 1 mAh cm^−2^ and (**b**) 10 mA cm^−2^ with 10 mAh cm^−2^. (**c**) Comparison of the current density, cycling capacity, and lifetime found in this study and that of other reported Zn/Zn cells with different additives. (**d**) EIS measurements of the Zn/Zn symmetrical cells before the cycling test. (**e**) Cyclic stability of Zn/Cu asymmetrical cells in the different electrolytes at 0.5 mA cm^−2^ with 1 mAh cm^−2^. (**f**–**h**) Corresponding discharge/charge profiles of Zn/Cu cells at different cycles.

The Zn/Zn symmetrical cells were fabricated to determine the influences of the stereoscopic configuration of sugar alcohol additive on the cyclic stability of the zinc electrode. Figure 3a presents that the Zn/Zn cell with the bare ZnSO_4_ electrolyte failed rapidly at 1 mA cm^−2^ and 1 mAh cm^−2^. In contrast, the Zn/Zn cells performed better cyclic performances when mannitol or sorbitol was added, especially the one with ZnSO_4_@M electrolyte cycled more than 1600 h. Additionally, the ZnSO_4_@M—containing cell displayed outstanding cycling stability for 900 h at the high test condition of 10 mA cm^−2^ (10 mAh cm^−2^), outperforming the cells with bare ZnSO_4_ and ZnSO_4_@S electrolytes, which cycled for about 85 h and 500 h, respectively (Figure 3b). The different degrees of improvement in the cyclic stability of the Zn anodes show that the stereoscopic configuration of the additive molecule has a significant effect on the cyclic stability of the Zn anode. The comparative data in Figure 3c and Tables S2 and S3 demonstrate that the zinc anode shows superior cycling stability and lower polarization voltage in the ZnSO_4_@M electrolyte, compared to the previously optimized electrolytes in other studies [11,12,34,35,40,43,49,50,51]. The results of electrochemical impedance spectroscopy (EIS) (Figure S7 and Figure 3d) further revealed much smaller charge—transfer resistances of the Zn/Zn symmetric cells with sugar alcohols. This indicated that the sugar alcohol additives accelerate electron transport, which helps homogenize the Zn^2+^ distribution in the Zn deposition process [51]. 

Since CE is a crucial factor in achieving the commercialization of AZIBs, the CEs of the Zn/Cu asymmetric cells utilizing bare ZnSO_4_, ZnSO_4_@M, and ZnSO_4_@S electrolytes were measured and are shown in Figure 3e [52]. The cells with bare ZnSO_4_ and ZnSO_4_@S failed after 108 cycles (average CE of 98.68%) and 114 cycles (average CE of 98.99%) at 0.5 mA cm^−2^ and 1 mAh cm^−2^, respectively. Remarkably, the Zn/Cu cell employing the ZnSO_4_@M electrolyte outperformed all others with an outstanding average CE of 99.66% sustained over 400 cycles. The voltage profiles in the ZnSO_4_ electrolytes without/with sugar alcohols during different cycles are compared in Figure 3f–h, where the zinc anode in the ZnSO_4_@M electrolyte showed high reversibility throughout 400 cycles. Compared to the ZnSO_4_@S electrolyte, the ZnSO_4_@M electrolyte significantly enhanced the Zn plating/stripping behaviors, likely due to the suitable stereoscopic configuration of the mannitol molecule, which promotes the reconstruction of the hydrated Zn^2+^—solvated sheath and the inhibition of dendritic growth and side reactions [53]. 

### 2.4. Zn Deposition Behavior

**Figure 4:**
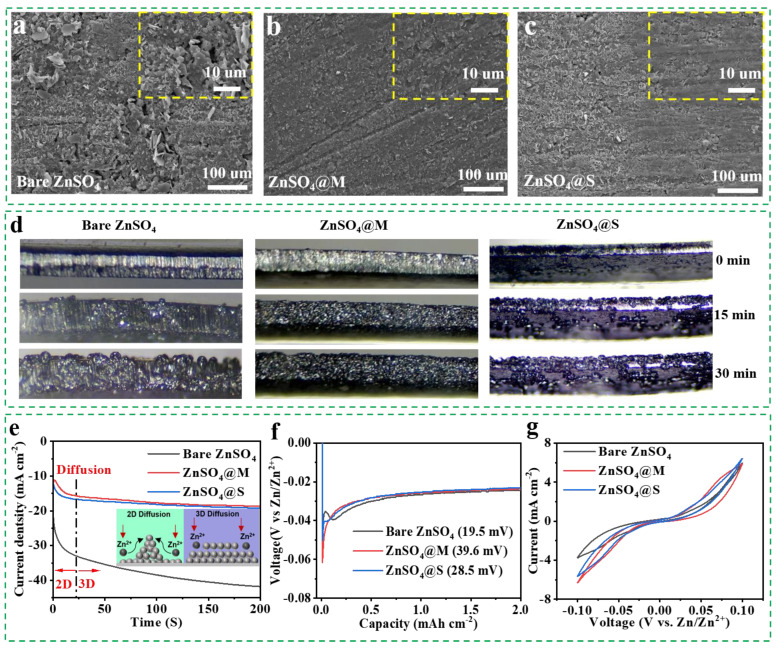
(**a**–**c**) Surface topography measurement of zinc foil substrates after cycling test in bare ZnSO_4_, ZnSO_4_@M, and ZnSO_4_@S electrolytes. (**d**) In situ optical microscopy images of the zinc electrode after 0, 15, and 30 min in bare ZnSO_4_, ZnSO_4_@M, and ZnSO_4_@S electrolytes. (**e**) CA measurement of Zn/Zn symmetrical cells with bare ZnSO_4_, ZnSO_4_@M, and ZnSO_4_@S electrolytes. (**f**) The initial Zn NOPs using the Zn/Cu half cells with various electrolytes. (**g**) CV curves of Zn/Zn symmetric cells with various electrolytes at 5 mV s^−1^.

To further examine the impact of sugar alcohols on the deposition evolution of Zn, the morphologies of zinc foil substrates were compared after 50 cycles. Inhomogeneous Zn deposition with obvious protuberances was observed on the morphology of the Zn foil (Figure 4a) in the bare ZnSO_4_ electrolyte. In contrast, Figure 4b,c demonstrates that the Zn deposition on the zinc foils in sugar alcohol—modified electrolytes is more uniform, especially in the ZnSO_4_@M electrolyte [54]. In addition, an in situ optical system was performed to investigate the zinc nucleation and deposition behavior (Figure 4d and Figure S8). Zn protrusions became visible with the increasing electrodeposition time in the bare ZnSO_4_ electrolyte, eventually inducing the zinc dendrite growth. In comparison, the topography of the Zn plate in the ZnSO_4_@M electrolyte was always dense and uniform during the continuous deposition, whereas the ZnSO_4_@S electrolyte resulted in small Zn protrusions and loose deposition [55]. 

The nucleation mechanism of Zn metal in ZnSO_4_ electrolytes with/without additive was confirmed through the chronoamperometry (CA) measurements [55,56]. Figure 4e exhibits that the zinc electrode in the bare ZnSO_4_ electrolyte displays a continuously decreased tendency of exchange current density within 200 s, corresponding to a prolonged and rampant two—dimensional (2D) diffusion behavior of zinc deposition, which causes the nonuniform Zn accumulation and vertical growth of dendrites. On the contrary, Zn foils in sugar alcohol—modified electrolytes displayed a constant three—dimensional (3D) diffusion behavior after a short 2D diffusion process for 25 s, indicating that the smooth, dense, and even Zn deposition was maintained over the 200 s duration [57]. The effect of sugar alcohol additives on Zn deposition aligned with findings from in situ optical photographs (Figure 4d) of Zn deposition. Moreover, the Zn nucleation process was further explored through the nucleation overpotential (NOP) analysis of Zn/Cu asymmetrical cells, as illustrated in Figure 4f. The initial NOPs of zinc electrodes in ZnSO_4_@M and ZnSO_4_@S electrolytes were found to be 39.6 mV and 28.5 mV, respectively, surpassing the NOP of the bare ZnSO_4_ electrolyte (19.5 mV). According to classical nucleation theory, a higher NOP encourages the formation of smaller and denser Zn nuclei, potentially leading to fine—grained deposits that result in a compact and uniform surface for subsequent Zn deposition [36]. Furthermore, CV curves with the sugar alcohol additive exhibited much stronger intensities of redox peaks (Figure 4g), indicating the enhanced electrochemical reactivity for Zn deposition [58].

### 2.5. Inhibition of Side Reactions

**Figure 5:**
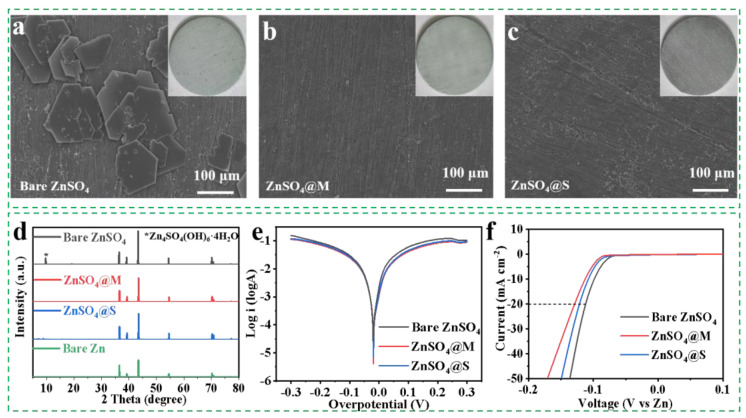
(**a**–**c**) Surface morphology images and optical photographs of zinc foils soaked in the ZnSO_4_ electrolytes with/without additive for one week. (**d**) XRD patterns of zinc electrodes in the ZnSO_4_ electrolytes with/without additive after cycling test. (**e**) Linear polarization curves in the ZnSO_4_ electrolytes with/without additive. (**f**) LSV curves in the ZnSO4 electrolytes with/without additive.

To verify the corrosion resistance of the sugar alcohols, the Zn foils were immersed in the bare ZnSO_4_, ZnSO_4_@M, and ZnSO_4_@S electrolytes for 7 days. Figure 5a–c show that the polygonal micro—flakes were distributed on the zinc foil surface in the bare ZnSO_4_ electrolyte, while no obvious by—products appeared on the surface of the zinc foils in the ZnSO_4_@M and ZnSO_4_@S electrolytes. The X-ray diffraction (XRD) pattern (Figure 5d) confirmed that the polygonal by—products observed in the bare ZnSO_4_ electrolyte were Zn_4_SO_4_(OH)_6_·4H_2_O induced by the corrosion reaction [59]. However, no undesired diffraction peaks of by—products were observed on the XRD profiles of Zn anodes in the ZnSO_4_ electrolytes with sugar alcohol additives, indicating the successful prevention of the corrosion reaction induced by H_2_O and SO_4_^2−^. Additionally, the corrosion currents (Figure 5e) remarkably decreased in ZnSO_4_@M and ZnSO_4_@S electrolytes, demonstrating the more efficient prevention of corrosion reaction by sugar alcohols [60]. Additionally, the potential for HER of electrolytes was evaluated using the linear sweep voltammetry (LSV) measurement, as illustrated in Figure 5f. The HER potential in the bare ZnSO_4_ electrolyte was recorded to be −112 mV at 20 mA cm^−2^, while it increased by 12 mV and 4 mV in the ZnSO_4_@M and ZnSO_4_@S electrolytes, respectively. This increase suggests that sugar alcohol additives have a more pronounced inhibitory of hydrogen evolution, leading to higher potentials in the modified electrolytes [61]. 

### 2.6. Working Mechanism of Additive

**Figure 6:**
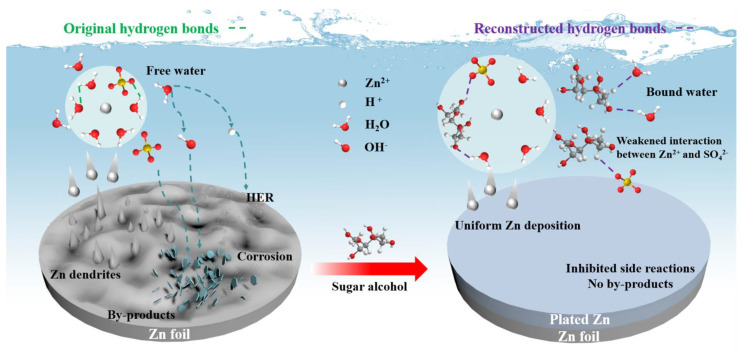
Schematic diagram of hydrated Zn^2+^—solvation structure at Zn anodes in the ZnSO_4_ electrolytes with/without additive.

Upon combining the experimental and theoretical results, a comprehensive understanding of the working mechanism of sugar alcohol additives can be attained. It is acknowledged that Zn^2+^ exists in the aqueous electrolyte as the [Zn(H_2_O)_x_]^2+^ solvation structure, where the central Zn^2+^ ions function as the Lewis acid site, hydrolyzing H_2_O to H^+^ and OH^−^ and inducing side reactions on the zinc anode surface in a mildly acidic environment. Additionally, dendrite growth is another frequent phenomenon during the repeated charge/discharge process (Figure 6). Herein, the introduction of sugar alcohol into the electrolyte alters the electrolyte coordination environment, leading to a partial replacement of H_2_O molecules in the solvation shell with sugar alcohol additives. Meanwhile, the added sugar alcohol molecules disrupt the existing hydrogen bond between H_2_O molecules in the electrolyte, allowing more free H_2_O molecules to be captured by sugar alcohol with hydroxyl groups, reducing the water molecules involved in interface side reactions, lowering proton activity, and thus mitigating parasitic reactions caused by free H_2_O. Furthermore, due to the regulation of the Zn^2+^—solvation sheath by sugar alcohol, the Zn nucleation process requires a slightly higher overpotential, resulting in a homogeneous nuclei distribution and even Zn deposition without dendrites. Notably, among the two sugar alcohol additives employed in this study, mannitol, owing to its more suitable stereoscopic configuration, is utilized as an additive for achieving a Zn anode with superior electrochemical performance.

### 2.7. Electrochemical Properties’ Characterization

**Figure 7:**
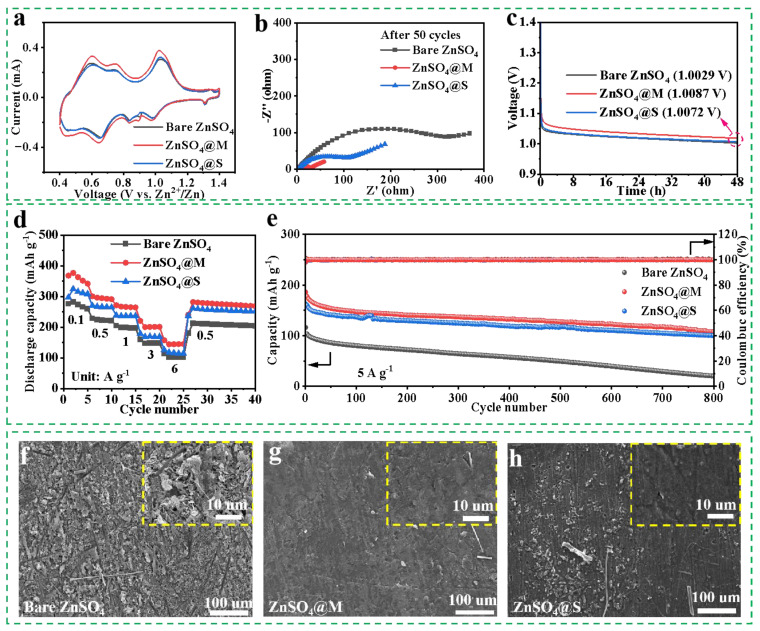
(**a**) CV curves of the Zn/NH_4_V_4_O_10_ full cells at 0.1 mV s^−1^. (**b**) Nyquist plots of the Zn/NH_4_V_4_O_10_ full cells after 50 cycles. (**c**) Time—dependent open—circuit voltages of the Zn/NH_4_V_4_O_10_ full cells for self—discharge tests. (**d**) Rate performances of the Zn/NH_4_V_4_O_10_ full cells measured from 0.1 to 6 A g^−1^. (**e**) Cyclic performances of the Zn/NH_4_V_4_O_10_ full cells at 5 A g^−1^. (**f**–**h**) Surface topography measurement of Zn electrodes at the 50th cycle in the Zn/NH_4_V_4_O_10_ full cells with various electrolytes.

To demonstrate the practical application of the sugar alcohol additives, full cells were assembled with an NH_4_V_4_O_10_ cathode (Figure S9) and a Zn anode [58]. Figure 7a exhibits the CV curves for the Zn/NH_4_V_4_O_10_ full cells. The full cell utilizing ZnSO_4_@M displayed a larger area compared to that with the bare ZnSO_4_ electrolyte, which corresponds to the aforementioned improved reaction kinetics [62]. The multi—cycle CV curves (Figure S10) overlapped well, which further elucidated the electrochemical stability of mannitol as an electrolyte additive. Additionally, as shown in Figure S11 and Figure 7b, the R_ct_ reduction in the cells using ZnSO_4_@M and ZnSO_4_@S further exemplifies the influences of sugar alcohols on inhibiting dendritic growth and adverse parasitic reactions on the zinc electrode. Another critical factor affecting the performance of AZIBs is their self—discharge characteristics, which can be assessed through the open—circuit voltage of the Zn/NH_4_V_4_O_10_ full cell after charging and standing for 48 h [63]. Figure 7c reveals that the initial open—circuit voltage (1.4 V) of the Zn/NH_4_V_4_O_10_ cells incorporated with ZnSO_4_@M or ZnSO_4_@S decays to 1.0087 V and 1.0072 V, respectively, while that of the bare cell drops to 1.0029 V. Moreover, Figure 7d shows a low rate performance of the Zn/NH_4_V_4_O_10_ cell cycling in the bare ZnSO_4_ electrolyte. This is likely due to the development of an insulating passivation layer on the zinc metal that hinders the interfacial transportation of Zn^2+^ [64,65,66]. On the other hand, the Zn/NH_4_V_4_O_10_ cells with ZnSO_4_@M and ZnSO_4_@S demonstrated exceptional cycling stability after 800 cycles at 5 A g^−1^, surpassing the performance of the bare ZnSO_4_ electrolyte. Meanwhile, unlike the unstable charge/discharge platforms and enlarged over—potential curves observed with the bare ZnSO_4_ electrolyte, the full cells containing sugar alcohols maintained stable voltage plateaus following multiple cycles without obvious capacity degradation, as illustrated in Figure S12. Furthermore, the morphology image (Figure 7f) of the cycled Zn anode demonstrates the dendrite growth in the bare ZnSO_4_ electrolyte, while Figure 7g,h shows smooth deposition without noticeable dendrite formation in the sugar alcohol—modified electrolytes. 

To further explore the universality of sugar alcohol additives, Zn/MnO_2_ full cells were assembled using a MnO_2_ cathode (Figure S13), and their lifetimes were investigated in different electrolytes, as illustrated in Figure S14. Notably, all sugar alcohol—modified cells exhibited superior capacity retention rates after 800 cycles compared to the bare cell, highlighting the broad applicability of sugar alcohol additives.

## 3. Experimental Section

### 3.1. Materials

Zinc sulfate heptahydrate (ZnSO_4_·7H_2_O), ammonium metavanadate (NH_4_VO_3_), oxalic acid (H_2_C_2_O_4_·2H_2_O), potassium permanganate (KMnO_4_), and 1-methyl-2-pyrrolidinone (NMP) were sourced from Shanghai Aladdin Biochemical Technology Co., Ltd., Shanghai, China. HCl was purchased from Luoyang Haohua Chemical Reagent Co. Ltd., Luoyang, China. Super P carbon was bought from Kluthe Chemical Industrial Co., Ltd., Shanghai, China. Polyvinylidene fluoride (PVDF) was bought from Guangdong Zhuguang New Energy Technology Co., Ltd., Guangdong, China. Mannitol and sorbitol were obtained from Sinopharm Chemical Reagent Co., Ltd., Shanghai, China. All materials were used as received. 

### 3.2. Preparation of Cathode

A 2 M ZnSO_4_ electrolyte was obtained by dissolving ZnSO_4_·7H_2_O in deionized (DI) water. Mannitol and sorbitol were, respectively, added to the prepared electrolyte to acquire the modified electrolytes.

### 3.3. Preparation of the Electrolyte

The NH_4_V_4_O_10_ material was prepared by the traditional hydrothermal method. Firstly, 1.170 g of NH_4_VO_3_ was added to 35 mL of DI water on a hot plate set to 80 °C and stirred to form a yellowish solution. Next, 1.891 g of H_2_C_2_O_4_·2H_2_O was slowly dissolved into the solution. Subsequently, the prepared solution was put into a 50 mL Teflon—lined stainless steel autoclave and placed in an oven at 140 °C for 48 h. The precipitates were then filtered and rinsed with DI water and desiccated at 60 °C for 12 h to obtain the NH_4_V_4_O_10_ powder. 

The MnO_2_ material was also prepared by the traditional hydrothermal method. A 6 mM quantity of potassium permanganate was dissolved in 60 mL DI water and then stirred evenly. Next, 20 mM HCl was added dropwise to the above solution, and after mixing and stirring, it was poured into a 50 mL Teflon—lined stainless steel autoclave, heated to 140 °C, and kept for 12 h. After the above product was naturally cooled to room temperature, the lower precipitate was washed three times with DI water and alcohol alternately and dried overnight in a vacuum oven to obtain the MnO_2_ powder.

The cathodes were fabricated by mixing NH_4_V_4_O_10_ (or MnO_2_), Super P carbon, and PVDF in a weight ratio of 7:2:1, and NMP was added drop by drop. This mixture was then coated on a stainless steel mesh and dried at 80 °C overnight. Lastly, the mesh was cut into round sheets with a diameter of 12 mm, and the mass loading was kept to around 1.7 mg cm^−2^.

### 3.4. Characterization

The structure and chemical bond were characterized by a FT—IR spectrometer (Shimadzu, IRTracer—100, Tokyo, Japan). A Raman microscope (Raman, LabRAM HR Evolution, Shanghai, China) with a 532 nm excitation laser line was used to analyze the H—bond network in the ZnSO_4_ electrolytes. The images revealing the surface morphology of the Zn anodes were acquired by field emission scanning electron microscopy (JSM—7800F, JEOL, Akishima, Japan). The in situ optical images of Zn deposition were collected with an optical microscope (DMSZ8, Ningbo, China). The structural and phase characteristics of the samples were recorded by an XRD diffractometer (Bruker D8 ADVANCE, Cu—kα, λ = 1.5418 Å).

### 3.5. Electrochemical Measurements

The symmetrical Zn/Zn cells, Zn/Cu asymmetric cells, Zn/NH_4_V_4_O_10_, and Zn/MnO_2_ full cells were assembled in CR2032 coin cells configuration by using 2 M ZnSO_4_ with or without sugar alcohol additive as the electrolyte and glass fiber as the separator. The cycling, rate, and CE performance of the cells were measured by the Neware battery testing system (BTS—5V20 mA, Shenzhen, China). The electrochemical impedance measurements (EIS) of Zn/Zn symmetric cells were measured across the range of 10^5^ Hz to 10^−2^ Hz with an AC amplitude of 5 mV at room temperature on an electrochemical workstation (CHI—660E, Shanghai, China). The specific ionic conductivity was determined using the following equation: σ = l/(R_b_*S), where *σ* (mS cm^−1^) is the ionic conductivity, *l* (cm) is the separator’s thickness, *R_b_* (Ω) is the bulk resistance, and S (cm^2^) is the separator’s area. The LSV measurements were taken using a three—electrode system, with stainless steel working electrodes, and Zn metal reference and counter electrodes, with a change in voltage of 5 mV s^−1^ from 0 V to −0.4 V. The equilibrium redox voltages were obtained by fitting Tafel plots for the Zn/Zn symmetric coin cells at 5 mV s^−1^ across voltages between −0.3 and 0.3 V. The CA measurements were carried out with a constant potential of 10 mV for 200 s to determine Zn deposition behavior. The performance of the CV was conducted at 1 mV s^−1^ across the voltage range between 0.4 and 1.4 V. 

### 3.6. Computational Details

DFT spin—polarized calculations were conducted using the DMol3 package, following the adoption of the generalized gradient approximation (GGA) in the Perdew—Burke—Ernzerhof form and the Semicore Pseudopotential method (DSPP) with the double numerical basis sets plus the polarization functional (DNP) [67,68,69]. To account for the dispersion interaction, a DFT—D correction with the Grimme scheme was applied [70]. SCF convergence for each electronic energy was set as 1.0 × 10^–6^ Ha, and the geometry optimization convergence criteria were established at 1.0 × 10^–6^ Ha for energy, 0.0001 Ha Å–1 for force, and 0.0001 Å for displacement, respectively. The investigation of energy barriers was accomplished through the linear and quadratic synchronous transit methods combined with the conjugated gradient refinement. Furthermore, the adsorption energies (*E_ads_*) were computed using *E_ads_ = E_ad/sub_* − *E_ad_* − *E_sub_*, where *E_ad/sub_*, *E_ad_*, and *E_sub_*, respectively, denote the total energies of the optimized adsorbate/substrate system, the adsorbate in the gas phase, and the clean substrate, respectively. The DFT had also been employed to describe the electrostatic potential (ESP). In this study, the B3LYP—D3(BJ)/6—31G(d,p) theoretical model has been adopted for the structural optimizations. To simulate the solvation effect, the self—consistent reaction field method with the polarized continuum model was utilized. Additionally, the ESP mapping was analyzed via the Multiwfn package 3.7 and VMD package 1.9.4 [71,72].

## 4. Conclusions

This study utilizes a combination of experimental analysis and theoretical calculations to compare the effects of mannitol and sorbitol, isomers used as additives in the ZnSO_4_ aqueous electrolyte. Mannitol, with its advantageous stereoscopic configuration, exhibits the strongest binding energy with Zn^2+^ and water molecules. This characteristic enables mannitol to coordinate efficiently with zinc ions, leading to modifications in solvation structure, reduced reactive water content within the electrolyte, and ultimately the prevention of zinc dendrite formation and undesirable side reactions. Consequently, Zn/Zn cells incorporating mannitol show superior long—term stability, with lifetimes extending to 1600 h at 1 mA cm^−2^ (1 mAh cm^−2^) and 900 h at 10 mA cm^−2^ (10 mAh cm^−2^). Furthermore, the inclusion of mannitol in Zn/NH_4_V_4_O_10_ and Zn/MnO_2_ full cells results in enhanced cycling performance, better rate capability, and improved capacity retention over extended periods. Thus, this research not only achieves a highly reversible and high—rate Zn anode, but also presents a novel avenue for designing electrolyte additives for aqueous Zn—ion batteries by investigating the stereoscopic configuration of additive molecules.

## Data Availability

The data presented in this study are available on request from the corresponding author.

## Supplementary Materials

The following supporting information can be downloaded at: https://www.mdpi.com/article/10.3390/molecules29143416/s1, Figure S1: The stereoscopic configurations of mannitol and sorbitol molecules; Figure S2: Long—term cycling performances of Zn/Zn cells in 2 M ZnSO_4_ electrolytes with various mannitol contents (0 mM, 10 mM, 20 mM, and 30 mM) at 1 mA cm^−2^ and 1 mAh cm^−2^; Figure S3: Optical photographs of ZnSO_4_ electrolytes with/without additives; Figure S4: Raman spectra of the different electrolytes in the range from 1500 cm^−1^ to 1800 cm^−1^; Figure S5: Multi—cycle CV curves of Zn/Zn symmetric cells with the ZnSO_4_@Melectrolyte; Figure S6: Different bonds in (a) Zn^2+^—5H_2_O—SO_4_^2−^ and (b) mannitol—Zn^2+^—4H_2_O–SO_4_^2−^; Figure S7: EIS measurements of the Zn/Zn cells before cycling test; Figure S8: The in situ optical microscopy system to observe the Zn anode during the Zn plating process; Figure S9: (a) Morphology image and (b) XRD pattern of NH_4_V_4_O_10_ powder; Figure S10: Multi—cycle CV curves of Zn/NH_4_V_4_O_10_ symmetric cells with the ZnSO_4_@M electrolyte; Figure S11: Nyquist plots of Zn/NH_4_V_4_O_10_ full cells before cycling test; Figure S12: Voltage profiles of the Zn/NH_4_V_4_O_10_ full cells at 5 A g^−1^ for different cycles; Figure S13: (a) Morphology image and (b) XRD pattern of MnO_2_ powder; Figure S14: Cycling performance of Zn/MnO_2_ full cells tested at the current density of 0.5A g^−1^; Table S1: Bond lengths of the solvation structure of Zn^2+^ with/without mannitol; Table S2: Comparison of this study with other previously reported cycling performances of symmetrical cells; Table S3: Comparison of this study with other previously reported polarization voltages of symmetrical cells at 100 h.
