# Supplementary material for: Suitable Stereoscopic Configuration of Electrolyte Additive Enabling Highly Reversible and High—Rate Zn Anodes
Source: Molecules. 2024 Jul 21;29(14):3416. doi: 10.3390/molecules29143416 (PMC11280124; doi:10.3390/molecules29143416)
Supplement: Supplementary file 1 [file molecules-29-03416-s001.zip › molecules-3087391-supplementary.pdf]

Supporting Information

Suitable Stereoscopic Configuration of Electrolyte Additive Enabling Highly Reversible and High-Rate Zn Anodes

Binrui Xu [1], Yong Liu [2,*], Bo Zhao [2,3], Haoming Li [2], Min Liu [1], Huanxiao Mai [1] and Quanan Li [2,4,*]

[1] School of Information Engineering, Henan University of Science and Technology, Luoyang 471023, China; 9906065@haust.edu.cn (B.X.); liuimin1086@163.com (M.L.); mhxgby@163.com (H.M.)

[2] School of Materials Science and Engineering, Provincial and Ministerial Coconstruction of Collaborative Innovation Center for Non−Ferrous Metal New Materials and Advanced Processing Technology, Henan University of Science and Technology, Luoyang 471023, China; bozhao123@haust.edu.cn (B.Z.); lihaoming546123@gmail.com (H.L.)

[3] Key Laboratory of Cluster Science of Ministry of Education, Beijing Key Laboratory of Photoelectronic/Electrophotonic Conversion Materials, School of Chemistry and Chemical Engineering, Beijing Institute of Technology, Beijing 100081, China

[4] Longmen Laboratory, Luoyang 471000, China

* Correspondence: liuyong209@haust.edu.cn (Y.L.); liquanan2016@163.com (Q.L.)

Tel.: +86−158−9664−9559 (Y.L.); +86-13837911662 (Q.L.)

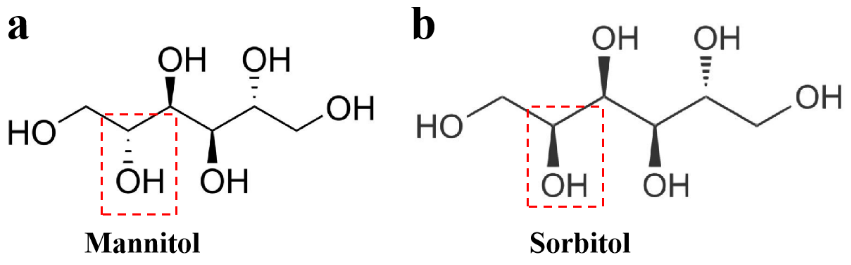

Figure S1. The stereoscopic configurations of mannitol and sorbitol molecules.

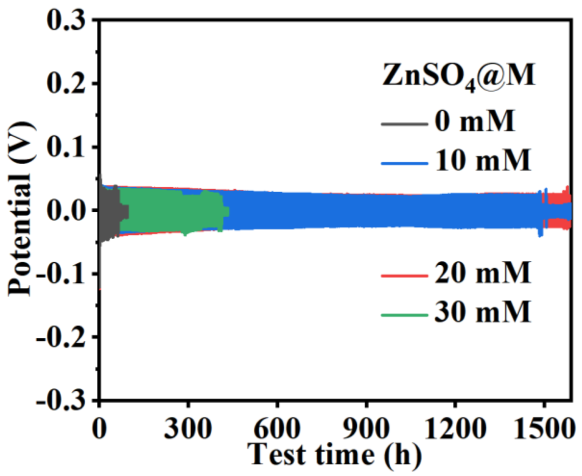

Figure S2. Long-term cycling performances of Zn/Zn cells in 2 M $ZnSO_4$ electrolytes with various mannitol contents (0 mM, 10 mM, 20 mM, and 30 mM) at 1 mA cm^{-2}, 1 mAh cm^{-2}.

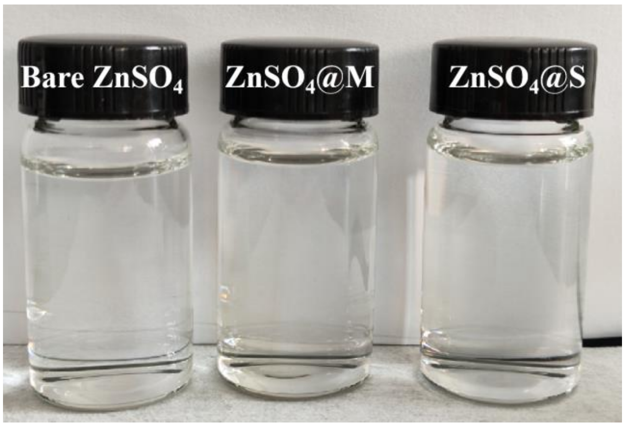

Figure S3. Optical photographs of $ZnSO_4$ electrolytes with/without additives.

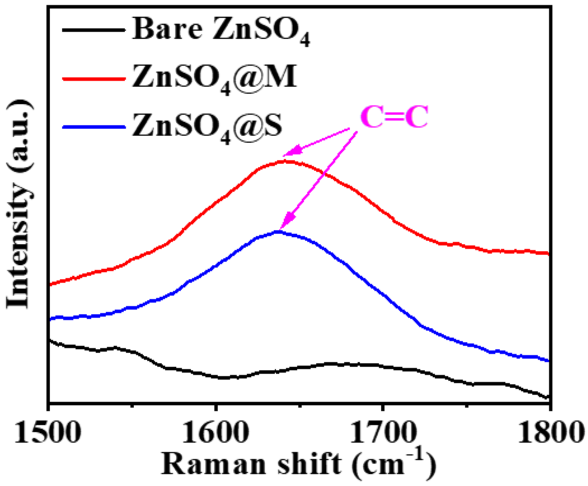

Figure S4. Raman spectra of the different electrolytes in the range from 1500 cm^{-1} to 1800 cm^{-1}.

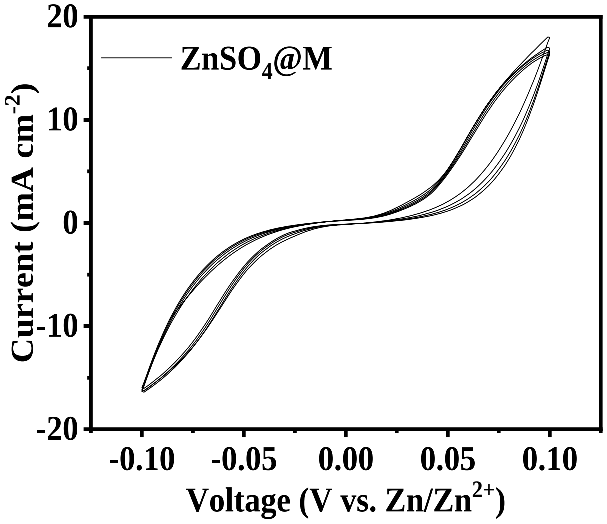

Figure S5. Multi-cycle CV curves of Zn/Zn symmetric cells with the $ZnSO_4$@M electrolyte.

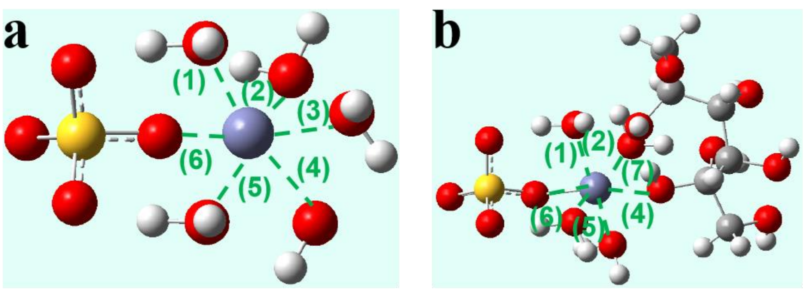

Figure S6. Different bonds in (a) Zn^{2+}-5H_2O-SO_4^{2-} and (b) mannitol-Zn^{2+}-4H_2O-SO_4^{2-}.

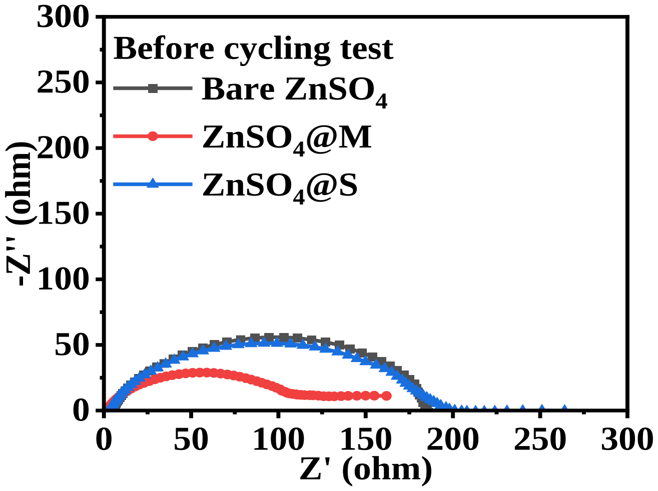

Figure S7. EIS measurements of the Zn/Zn cells before cycling test.

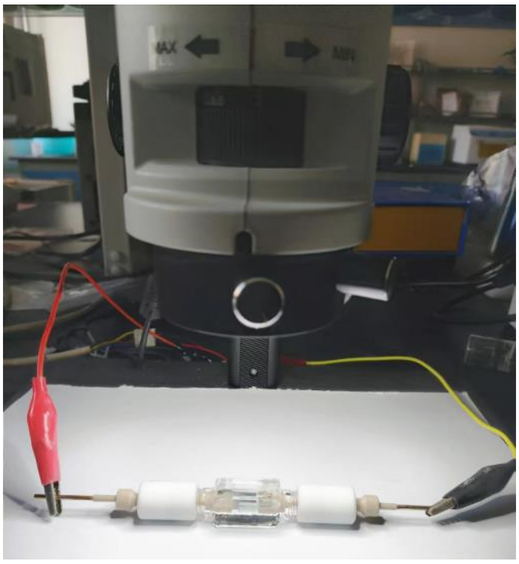

Figure S8. The in-situ optical microscopy system to observe the Zn anode during the Zn plating process.

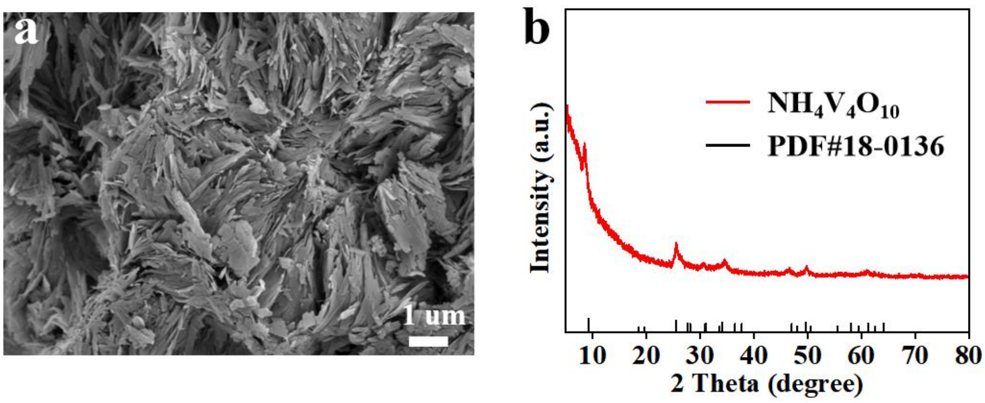

Figure S9. (a) Morphology image and (b) XRD pattern of $NH_4V_4O_{10}$ powder.

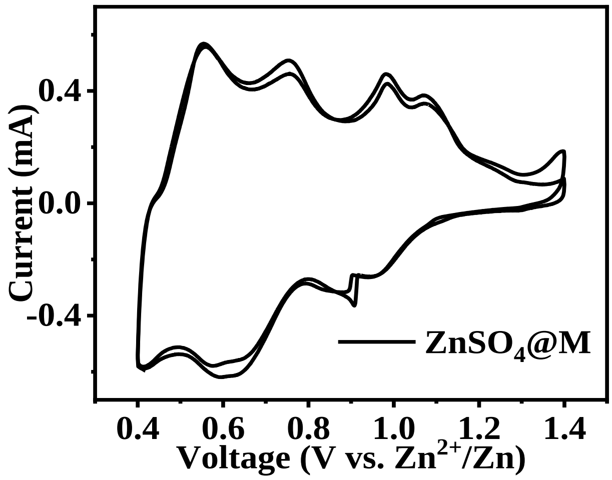

Figure S10. Multi-cycle CV curves of Zn/$NH_4V_4O_{10}$ symmetric cells with the $ZnSO_4$@M electrolyte.

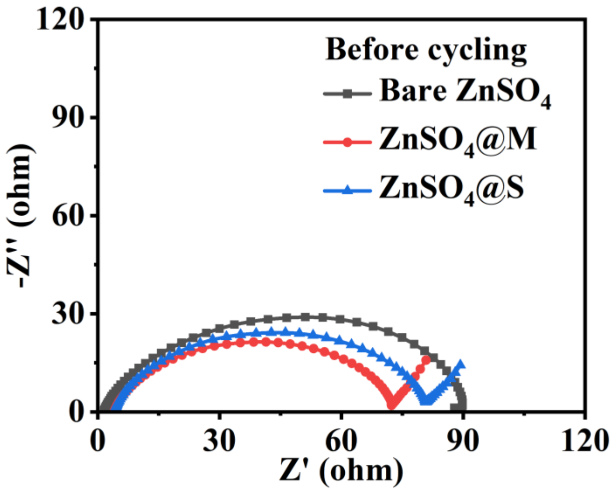

Figure S11. Nyquist plots of Zn/$NH_4V_4O_{10}$ full cells before cycling test.

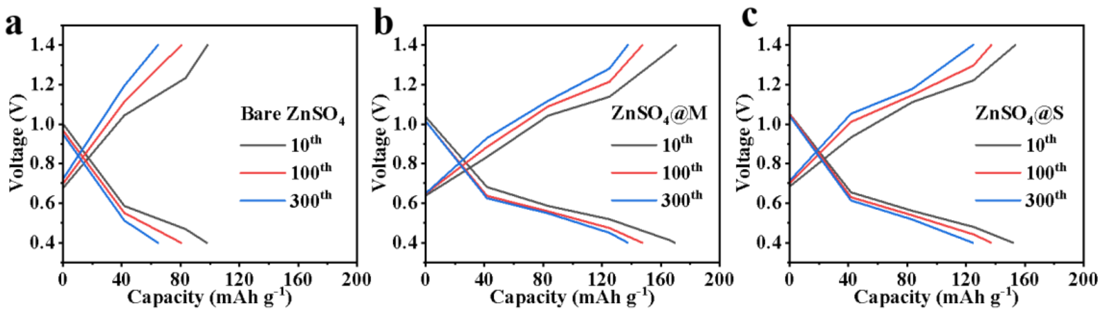

Figure S12. Voltage profiles of the Zn/$NH_4V_4O_{10}$ full cells at 5 A g^{-1} for different cycles.

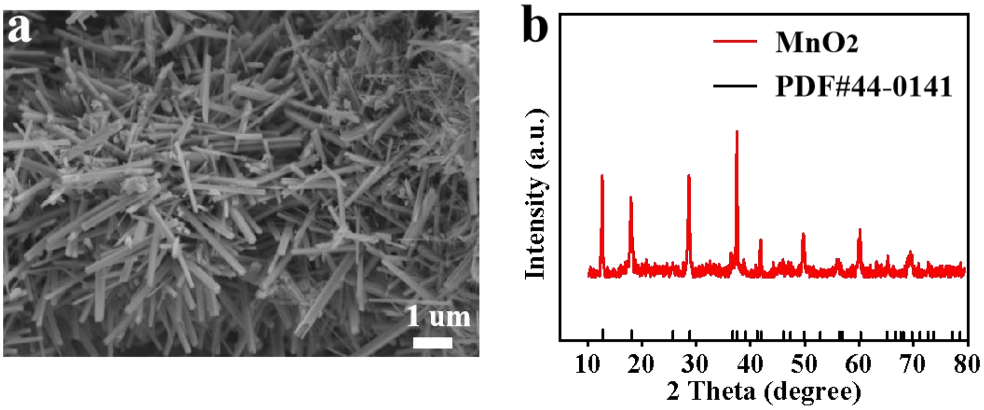

Figure S13. (a) Morphology image and (b) XRD pattern of MnO_2 powder.

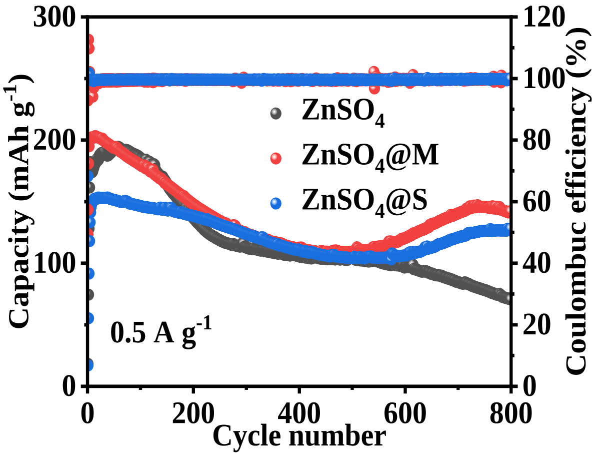

Figure S14. Cycling performance of Zn/MnO_2 full cells tested at the current density of 0.5 A g^{-1}.

Table S1. Bond lengths of the solvation structure of Zn^{2+} with/without mannitol.

**Bond**	**Bond length (Å) in Zn^{2+}-$5H_2O$-SO_4^{2-}**	**Bond length (Å) in mannitol-Zn^{2+}-$4H_2O$-SO_4^{2-}**
Zn^{2+}-H_2O (1)	2.10	2.12
Zn^{2+}-H_2O (2)	2.18	2.07
Zn^{2+}-H_2O (3)	2.07	None
Zn^{2+}-H_2O (4)	2.15	2.14
Zn^{2+}-H_2O (5)	2.12	2.08
Zn^{2+}-SO_4^{2-} (6)	2.03	2.02
Zn^{2+}-mannitol (7)	None	2.27
Average Zn^{2+}-H_2O	2.12	2.10

Table S2. Comparison of this work with other previously reported cycling performances of symmetrical cells.

Additive	Areal capacity (mAh cm^{-2})	Current density (mA cm^{-2})	Cycle life (hour)	Reference
mannitol	1	1	1600	This work
mannitol	10	10	900	This work
TBA_2SO_4	2	2	300	[1]
NMP	1	1	540	[2]
Gly	4	2	600	[3]
Arg	4	10	900	[4]
Xylitol	1	1	1100	[5]
NSQDs	0.5	2	1300	[6]

Table S3. Comparison of this work with other previously reported polarization voltages of symmetrical cells at 100 h.

Additive	Current density (mA cm^{-2})	Areal capacity (mAh cm^{-2})	Polarization voltage (mV)	Reference
mannitol	1	1	17	This work
2,4-DHP	1	1	18	[7]
Urea	1	1	19	[8]
Dopamine	1	1	41	[9]
Arg	1	1	49	[4]
Gly	1	1	53	[3]
NMP	1	1	55	[2]

References

1. A. Bayaguud, X. Luo, Y. Fu, C. Zhu, Cationic Surfactant-Type Electrolyte Additive Enables Three-Dimensional Dendrite-Free Zinc Anode for Stable Zinc-Ion Batteries, ACS Energy Lett. 5(9) (2020) 30123020, https://doi.org/10.1021/acsenergylett.0c01792.
2. T.C. Li, Y. Lim, X.L. Li, S. Luo, C. Lin, D. Fang, S. Xia, Y. Wang, H.Y. Yang, A Universal Additive Strategy to Reshape Electrolyte Solvation Structure toward Reversible Zn Storage, Adv. Energy Mater. 12(15) (2022) 2103231, https://doi.org/https://doi.org/10.1002/aenm.202103231.
3. Q. Gou, H. Luo, Q. Zhang, J. Deng, R. Zhao, O. Odunmbaku, L. Wang, L. Li, Y. Zheng, J. Li, D. Chao, M. Li, Electrolyte Regulation of Bio-Inspired Zincophilic Additive toward High-Performance Dendrite-Free Aqueous Zinc-Ion Batteries, Small 19(10) (2023) 2207502, https://doi.org/https://doi.org/10.1002/smll.202207502.
4. H. Lu, X. Zhang, M. Luo, K. Cao, Y. Lu, B.B. Xu, H. Pan, K. Tao, Y. Jiang, Amino Acid-Induced Interface Charge Engineering Enables Highly Reversible Zn Anode, Adv. Funct. Mater. 31(45) (2021) 2103514, https://doi.org/https://doi.org/10.1002/adfm.202103514.
5. H. Wang, W. Ye, B. Yin, K. Wang, M.S. Riaz, B.-B. Xie, Y. Zhong, Y. Hu, Modulating Cation Migration and Deposition with Xylitol Additive and Oriented Reconstruction of Hydrogen Bonds for Stable Zinc Anodes, Angew. Chem., Int. Ed. 62(10) (2023) e202218872, https://doi.org/https://doi.org/10.1002/anie.202218872.
6. F. Wang, H. Lu, H. Zhu, L. Wang, Z. Chen, C. Yang, Q.-H. Yang, Mitigating the interfacial concentration gradient by negatively charged quantum dots toward dendrite-free Zn anodes, Energy Storage Mater. 58 (2023) 215-221, https://doi.org/https://doi.org/10.1016/j.ensm.2023.03.032.
7. B. Xu, G. Wang, Y. Liu, Q. Li, F. Ren, J. Ma, Co-regulation effect of solvation and interface of pyridine derivative enabling highly reversible zinc anode, Journal of Materials Science & Technology 204 (2025) 1-9, https://doi.org/https://doi.org/10.1016/j.jmst.2024.03.024.
8. B.-R. Xu, Q.-A. Li, Y. Liu, G.-B. Wang, Z.-H. Zhang, F.-Z. Ren, Urea-induced interfacial engineering enabling highly reversible aqueous zinc-ion battery, Rare Metals 43(4) (2024) 1599-1609, https://doi.org/10.1007/s12598-023-02541-4.
9. X. Zeng, K. Xie, S. Liu, S. Zhang, J. Hao, J. Liu, W.K. Pang, J. Liu, P. Rao, Q. Wang, J. Mao, Z. Guo, Bio-inspired design of an in situ multifunctional polymeric solid–electrolyte interphase for Zn metal anode cycling at 30 mA cm−2 and 30 mA h cm−2, Energy Environ. Sci 14(11) (2021) 5947-5957, https://doi.org/https://doi.org/10.1039/D1EE01851E.
